# Global burden and forecast of malignant neoplasms of bone and articular cartilage from 1990 to 2030

**DOI:** 10.3389/fmed.2025.1697423

**Published:** 2025-11-17

**Authors:** Su Wu, Yun Wu, Jun Huang, Ning Yan

**Affiliations:** 1 Department of Orthopedics, The Third People's Hospital of Jingdezhen, Jingdezhen, Jiangxi, China; 2 Department of Nephrology, The Third People's Hospital of Jingdezhen, Jingdezhen, Jiangxi, China; 3 Department of Orthopedics, Yichun Hospital of Traditional Chinese Medicine, Yichun, Jiangxi, China; 4 Department of Orthopedics, Shanghai Institute of Bone Tumor, Shanghai Tenth People's Hospital, Tongji University School of Medicine, Shanghai, China

**Keywords:** malignant neoplasms of bone and articular cartilage, social-demographic index, prediction model, burden of disease, epidemiology

## Abstract

**Background:**

Malignant neoplasms of bone and articular cartilage (MNBAC) are rare but highly lethal cancers, disproportionately affecting children and adolescents. Despite their clinical significance, comprehensive global assessments of their burden and long-term trends remain scarce.

**Methods:**

Using Global Burden of Disease (GBD) 2021 data (1990–2021), we estimated age-standardized incidence (ASIR), death (ASDR), and disability-adjusted life years (DALY ASR) across 21 geographic and 5 sociodemographic index (SDI) regions. Temporal trends were quantified with estimated annual percentage change (EAPC). Polynomial regression across 1990–2021 was used to assess the association between SDI and disease burden. Age–sex stratification, age–period–cohort (APC) modeling, and Bayesian APC (BAPC) projections to 2030 were conducted.

**Results:**

Globally, the MNBAC burden modestly increased over three decades. High-SDI regions experienced declining trends, whereas middle- and low-middle-SDI regions showed substantial increases in incidence and mortality. The polynomial regression analysis revealed that there was an overall negative correlation between SDI and disease burden, but a significant nonlinear relationship existed. Adolescents and older adults carried the highest incidence, with males more frequently affected. Cohort analyses revealed increasing risks in middle-SDI populations, and projections indicate a continuing rise in burden in these regions by 2030, while stabilization or decline is expected elsewhere.

**Conclusion:**

Although rare, MNBAC imposes a growing global health burden, particularly in middle-SDI regions and vulnerable populations. Recognizing the nonlinear link between socioeconomic development and disease burden highlights the need for stage-specific strategies, including strengthening early diagnosis, equitable access to care, and targeted prevention strategies is critical to reducing future disparities.

## 1 Introduction

Malignant neoplasms of bone and articular cartilage (MNBAC) are rare but highly aggressive mesenchymal tumors that contribute disproportionately to global disability and premature mortality. In 2020, the World Health Organization (WHO) published the fifth edition of the *Classification of Tumours* series, regarded as the gold standard in oncological diagnostics. Volume 3 of this edition, focusing on soft tissue and bone tumors, classifies MNBAC within the category of other malignant neoplasms, accounting for approximately 0.2% of all cancers worldwide. Although uncommon, their clinical significance is magnified by poor treatment outcomes and the frequent occurrence of skeletal complications. For instance, bone metastases are reported in 6.9% of patients within 5 years after solid tumor diagnosis, rising to 8.4% at 10 years, underscoring the challenges of managing advanced bone malignancies where therapeutic options remain limited and prognosis is poor (1, 2). Consequently, targeted prevention, early detection, and timely intervention are essential priorities for orthopedic oncology and global cancer control.

The Global Burden of Disease (GBD) study has emerged as a cornerstone for quantifying disease burden across 204 countries and territories. Through standardized methodology and periodic updates, the GBD provides comparable estimates of incidence, mortality, and disability-adjusted life years (DALYs), facilitating cross-disease and cross-regional analyses (3, 4). These estimates are vital for monitoring epidemiological transitions, prioritizing health resources, and guiding evidence-based policy. Recent GBD analyses have highlighted evolving patterns in musculoskeletal and cancer-related conditions. For example, although age-standardized incidence rates (ASIRs) of certain fractures have declined, years lived with disability (YLDs) continue to rise, illustrating the growing burden of survivable yet disabling diseases. Regional investigations, such as those on extremity fractures in the Middle East and North Africa, further underscore the utility of the GBD framework in capturing geographically nuanced disease dynamics (5).

Nevertheless, rare but clinically important conditions like MNBAC remain underrepresented in global estimates. These tumors are frequently subsumed within broader musculoskeletal or cancer categories, obscuring their distinct epidemiological features. Consequently, existing data are often fragmented, outdated, or geographically constrained, limiting our ability to understand the true global and sociodemographic heterogeneity of MNBAC and hindering the development of targeted prevention and treatment strategies (6, 7).

To bridge this knowledge gap, the present study provides a comprehensive analysis of MNBAC burden from 1990 to 2021 at global, regional, and national levels. We assess trends across sociodemographic index (SDI) regions, age, sex, and geography, and apply Bayesian age–period–cohort modeling to forecast incidence, mortality, and DALYs through 2030. By integrating robust epidemiological indicators with predictive modeling, this study aims to inform targeted cancer control strategies and support evidence-based policy formulation worldwide.

## 2 Methods

### 2.1 Data source

The Global Burden of Disease (GBD) study integrates data from multiple sources, including vital registration systems, surveys, and hospital records. To address data uncertainty and potential under-ascertainment, particularly in low-resource settings, GBD employs statistical modeling techniques, such as the Cause of Death Ensemble Model (CODEm) and predictive algorithms to impute missing values (8). Uncertainty intervals are generated for all estimates to reflect the combined effects of sampling error, measurement error, and model assumptions. These measures help to provide more reliable estimates even where direct data are sparse, though limitations remain in regions with very limited reporting.

Data on malignant neoplasms of bone and articular cartilage (MNBAC) were obtained from the GBD 2021 study, covering the period 1990–2021 (9). Within the GBD framework, MNBAC is defined according to the International Classification of Diseases (ICD). Specifically, ICD-10 codes C40–C41 (malignant neoplasms of bone and articular cartilage of limbs, other, and unspecified sites) and corresponding ICD-9 codes 170–170.9 were included. These codes were systematically mapped using the Cause of Death Ensemble Model (CODEm) and DisMod-MR 2.1 to ensure comparability across locations and years (10).

### 2.2 Temporal trend analysis

Long-term trends in age-standardized incidence rate (ASIR), age-standardized death rate (ASDR), and age-standardized disability-adjusted life years (DALY ASR) from 1990 to 2021 were assessed across 21 GBD regions and five sociodemographic index (SDI) quintiles. Trends were quantified using the estimated annual percentage change (EAPC), derived from a log-linear regression model:


ln(y)=α+βx+ε

where *y* is the age-standardized rate, *x* is calendar year, and *β* is the slope. The EAPC and its 95% confidence interval (CI) were calculated to determine whether trends were increasing or decreasing.

### 2.3 Correlation with socio-demographic index

To comprehensively assess the relationship between socioeconomic development and disease burden, we extended our correlation analysis to include all available data from 1990 to 2021 across 204 countries and territories, rather than limiting the analysis to a single cross-sectional time point. The association between national SDI values and the ASIR, ASDR, and DALY ASR was examined using polynomial regression models. Regression coefficients, coefficients of determination (R^2^), and corresponding *p*-values were calculated to evaluate the strength and statistical significance of these associations over time.

### 2.4 Age- and sex-stratified analysis

Incidence, mortality, and DALYs were stratified by sex (male and female) and by 19 five-year age groups ranging from <5 years to ≥95 years, in order to identify age- and sex-specific patterns of disease burden.

### 2.5 Age-period-cohort (APC) analysis

To distinguish between time effects and cohort effects, we used the APC model in the NCI Age-Period-Cohort network tool to process the data^1^ (11). The data was divided into 20 age categories (from <5 years old to ≥95 years old), 6 5-year time periods (from 1992 to 1996 to 2017 to 2021), and corresponding birth cohorts (from 1900 to 1904 to 2015 to 2019). To ensure consistent five-year intervals, the 1990–1991 interval was excluded, and 1992 was selected as the starting point. Based on the NCI APC network tool, the period from 2002 to 2006 and the cohort from 1955 to 1959 were determined as the reference categories.

### 2.6 Bayesian age-period-cohort (BAPC) forecasting

Future burden was projected using a Bayesian APC model to estimate ASIR, ASDR, and DALY ASR from 2022 to 2030 globally and across SDI quintiles. Integrated nested Laplace approximation (INLA) was applied to obtain posterior distributions. Counts of incidence, deaths, and DALYs were projected by applying predicted rates to GBD population forecasts (12). The BAPC model flexibly captures age-, period-, and cohort-specific trends in MNBAC burden, it also inherently integrates uncertainty quantification through Bayesian posterior distributions, thereby aligning with the GBD uncertainty framework and ensuring consistency across our analyses.

### 2.7 Statistical analysis

All analyses were conducted using R software (version 4.5.1) and the NCI APC web tool. All statistical tests were two-sided, with *p* < 0.05 considered statistically significant.

## 3 Results

### 3.1 Global burden of MNBAC, 1990–2021

In 2021, there were an estimated 91,375 new cases of MNBAC worldwide, representing a 96.2% increase compared with 1990. During the same period, the number of deaths reached 66,114, an 85.9% increase, while DALYs increased by 60.0% to 2,525,828. From 1990 to 2021, global age-standardized rates all increased: ASIR (EAPC: 0.59; 95% CI: 0.51–0.68), ASDR (EAPC: 0.11; 95% CI: 0.02–0.21), and DALYs ASR (EAPC: 0.08; 95% CI: 0.00–0.17) (Table 1). Trends varied across SDI quintiles. Significant increases were observed in middle SDI regions (ASIR EAPC: 1.63; ASDR: 1.04; DALY ASR: 0.84) and low-middle SDI regions (ASIR: 0.53; ASDR: 0.29; DALY ASR: 0.22). In contrast, high SDI regions showed consistent declines across all indicators (ASIR: −0.31; ASDR: −1.05; DALY ASR: −1.01), as did low SDI regions. High-middle SDI regions exhibited mixed results, with decreases in ASDR and DALY ASR but no clear trend for ASIR. At the regional level, the largest increases in DALY ASR were seen in East Asia (EAPC: 1.77), followed by Southeast Asia, Central Latin America, and Oceania. ASDR rose significantly in six regions, led by East Asia (EAPC: 2.11). ASIR increased in nine regions, with East Asia showing the steepest rise (EAPC: 3.25), followed by Southeast Asia and Central Latin America.

**Table 1:** MNBAC DALYs ASR ASDR and ASIR in 1990 and 2021, and EAPC from 1990 to 2021 in global, 5 SDI regions, and 21 GBD regions.

Age-standardized mortality rates per 100 000	DALYs (Disability-Adjusted Life Years)			Deaths			Incidence		
Location_name	1990 [95%CI]	2021 [95%CI]	EAPC [95%CI]	1990 [95%CI]	2021 [95%CI]	EAPC [95%CI]	1990 [95%CI]	2021 [95%CI]	EAPC [95%CI]
Global	31.03 [36.86, 27.79]	31.06 [35.03, 25.44]	0.08 [0.00, 0.17]	0.79 [0.93, 0.73]	0.79 [0.89, 0.64]	0.11 [0.02, 0.21]	0.97 [1.13, 0.89]	1.11 [1.25, 0.90]	0.59 [0.51, 0.68]
High SDI	39.52 [49.89, 31.69]	33.32 [38.04, 26.15]	−1.01 [−1.12, −0.90]	0.54 [0.56, 0.52]	0.40 [0.43, 0.37]	−1.05 [−1.17, −0.93]	0.90 [0.93, 0.87]	0.83 [0.88, 0.78]	−0.31 [−0.40, −0.22]
High-middle SDI	28.01 [36.81, 24.76]	35.63 [47.96, 28.47]	−0.98 [−1.12, −0.84]	1.03 [1.16, 0.96]	0.78 [0.95, 0.56]	−0.84 [−0.99, −0.70]	1.31 [1.46, 1.22]	1.26 [1.52, 0.91]	0.08 [−0.06, 0.22]
Low SDI	33.27 [38.98, 25.58]	27.15 [32.67, 19.88]	−0.49 [−0.56, −0.42]	0.94 [1.15, 0.75]	0.85 [1.14, 0.69]	−0.44 [−0.52, −0.37]	1.03 [1.27, 0.82]	0.98 [1.31, 0.79]	−0.27 [−0.35, −0.19]
Low-middle SDI	37.23 [42.21, 34.68]	35.39 [42.50, 29.17]	0.22 [0.19, 0.25]	0.81 [0.94, 0.64]	0.89 [1.05, 0.72]	0.29 [0.26, 0.31]	0.90 [1.05, 0.70]	1.05 [1.26, 0.86]	0.53 [0.51, 0.55]
Middle SDI	22.32 [22.98, 21.59]	16.75 [17.59, 15.69]	0.84 [0.62, 1.07]	0.75 [1.00, 0.65]	0.94 [1.08, 0.73]	1.04 [0.78, 1.30]	0.84 [1.11, 0.73]	1.24 [1.43, 0.96]	1.63 [1.41, 1.85]
Andean Latin America	46.39 [54.98, 38.04]	32.49 [41.65, 25.40]	−1.16 [−1.24, −1.09]	1.15 [1.34, 0.96]	0.88 [1.1, 0.69]	−0.91 [−1.00, −0.83]	1.32 [1.56, 1.08]	1.14 [1.45, 0.89]	−0.46 [−0.54, −0.38]
Australasia	24.39 [25.81, 22.92]	14.29 [15.84, 12.77]	−1.83 [−2.22, −1.44]	0.57 [0.60, 0.54]	0.32 [0.35, 0.29]	−1.87 [−2.18, −1.56]	1.04 [1.10, 0.98]	0.75 [0.83, 0.68]	−1.15 [−1.49, −0.81]
Caribbean	35.72 [41.59, 31.74]	35.54 [42.81, 29.46]	0.05 [−0.08, 0.18]	0.95 [1.06, 0.86]	1.01 [1.17, 0.86]	0.18 [0.03, 0.33]	1.12 [1.27, 1.01]	1.27 [1.47, 1.07]	0.38 [0.22, 0.54]
Central Asia	33.49 [38.10, 29.67]	35.02 [40.40, 30.26]	0.18 [−0.02, 0.39]	0.88 [1.00, 0.76]	0.93 [1.07, 0.81]	0.21 [0.01, 0.42]	1.02 [1.17, 0.90]	1.16 [1.34, 1.01]	0.51 [0.29, 0.73]
Central Europe	48.42 [52.33, 44.11]	21.54 [23.75, 19.64]	−2.76 [−2.95, −2.58]	1.30 [1.41, 1.18]	0.63 [0.69, 0.57]	−2.55 [−2.74, −2.37]	1.63 [1.78, 1.49]	0.97 [1.06, 0.88]	−1.81 [−2.03, −1.59]
Central Latin America	29.12 [30.12, 28.13]	32.02 [35.35, 28.83]	0.62 [0.33, 0.91]	0.81 [0.83, 0.77]	0.87 [0.96, 0.78]	0.48 [0.20, 0.77]	0.90 [0.93, 0.86]	1.12 [1.24, 1.00]	1.03 [0.76, 1.31]
Central Sub-Saharan Africa	29.43 [38.82, 21.16]	24.70 [36.91, 15.49]	−0.60 [−0.71, −0.50]	0.80 [1.05, 0.56]	0.66 [0.97, 0.43]	−0.67 [−0.78, −0.56]	0.82 [1.09, 0.58]	0.70 [1.04, 0.45]	−0.56 [−0.68, −0.44]
East Asia	20.53 [35.34, 13.76]	29.38 [38.34, 18.80]	1.77 [1.16, 2.39]	0.59 [1.02, 0.40]	0.92 [1.21, 0.58]	2.11 [1.46, 2.77]	0.66 [1.14, 0.45]	1.40 [1.83, 0.89]	3.25 [2.65, 3.85]
Eastern Europe	61.28 [63.02, 59.44]	21.50 [23.40, 19.57]	−4.16 [−4.44, −3.89]	1.58 [1.62, 1.52]	0.52 [0.56, 0.47]	−4.46 [−4.74, −4.17]	2.12 [2.19, 2.04]	0.86 [0.94, 0.79]	−3.61 [−3.89, −3.33]
Eastern Sub-Saharan Africa	58.02 [77.73, 47.34]	50.59 [77.30, 36.59]	−0.65 [−0.72, −0.58]	1.39 [1.85, 1.13]	1.22 [1.83, 0.9]	−0.63 [−0.70, −0.56]	1.51 [2.00, 1.24]	1.38 [2.10, 1.02]	−0.46 [−0.53, −0.39]
High-income Asia Pacific	17.91 [19.86, 15.86]	10.44 [11.36, 9.67]	−1.85 [−2.01, −1.69]	0.38 [0.41, 0.34]	0.22 [0.24, 0.2]	−1.77 [−1.94, −1.60]	0.71 [0.77, 0.65]	0.54 [0.59, 0.50]	−0.94 [−1.07, −0.81]
High-income North America	18.69 [19.03, 18.31]	18.77 [19.48, 18.00]	−0.10 [−0.19, 0.00]	0.44 [0.45, 0.42]	0.44 [0.46, 0.42]	−0.11 [−0.24, 0.03]	0.82 [0.84, 0.80]	0.92 [0.96, 0.87]	0.26 [0.17, 0.35]
North Africa and Middle East	32.75 [38.77, 24.98]	29.72 [37.30, 24.02]	−0.25 [−0.28, −0.22]	0.84 [1.02, 0.66]	0.79 [1.01, 0.65]	−0.14 [−0.18, −0.10]	0.96 [1.15, 0.75]	1.06 [1.33, 0.87]	0.43 [0.38, 0.47]
Oceania	17.17 [30.92, 9.09]	19.27 [37.91, 7.65]	0.32 [0.15, 0.50]	0.42 [0.76, 0.23]	0.47 [0.90, 0.20]	0.33 [0.15, 0.51]	0.46 [0.82, 0.25]	0.52 [0.98, 0.22]	0.37 [0.19, 0.55]
South Asia	32.89 [39.27, 24.02]	32.57 [41.01, 26.68]	−0.11 [−0.17, −0.06]	0.77 [0.90, 0.58]	0.78 [0.98, 0.64]	−0.04 [−0.10, 0.03]	0.86 [1.02, 0.63]	0.94 [1.19, 0.77]	0.25 [0.17, 0.32]
Southeast Asia	31.03 [36.27, 23.33]	40.50 [50.60, 26.94]	1.01 [0.87, 1.16]	0.83 [0.97, 0.65]	1.14 [1.43, 0.76]	1.23 [1.07, 1.38]	0.90 [1.05, 0.70]	1.33 [1.65, 0.89]	1.44 [1.30, 1.58]
Southern Latin America	49.53 [54.68, 44.33]	27.59 [29.96, 25.41]	−1.83 [−2.05, −1.60]	1.35 [1.47, 1.21]	0.73 [0.80, 0.68]	−1.93 [−2.15, −1.71]	1.60 [1.74, 1.44]	1.07 [1.15, 0.99]	−1.25 [−1.47, −1.03]
Southern Sub-Saharan Africa	27.47 [32.66, 19.57]	25.78 [34.67, 21.36]	−0.40 [−0.70, −0.10]	0.73 [0.87, 0.51]	0.68 [0.89, 0.57]	−0.41 [−0.70, −0.12]	0.79 [0.94, 0.56]	0.77 [1.02, 0.64]	−0.32 [−0.58, −0.05]
Tropical Latin America	44.51 [46.46, 42.09]	36.40 [38.33, 34.73]	−0.42 [−0.52, −0.31]	1.26 [1.31, 1.19]	1.00 [1.05, 0.94]	−0.49 [−0.61, −0.36]	1.36 [1.43, 1.29]	1.24 [1.32, 1.18]	−0.04 [−0.15, 0.07]
Western Europe	29.01 [29.79, 28.15]	17.87 [18.72, 16.84]	−1.67 [−1.85, −1.50]	0.73 [0.75, 0.70]	0.44 [0.46, 0.41]	−1.70 [−1.88, −1.53]	1.24 [1.28, 1.20]	0.96 [1.01, 0.89]	−0.92 [−1.05, −0.79]
Western Sub-Saharan Africa	28.74 [36.98, 21.55]	27.29 [35.26, 20.20]	−0.16 [−0.22, −0.09]	0.66 [0.85, 0.48]	0.62 [0.80, 0.48]	−0.22 [−0.26, −0.17]	0.78 [0.99, 0.58]	0.79 [1.01, 0.59]	0.06 [−0.01, 0.12]

Red indicates an increase (EAPC > 0), blue indicates a decrease (EAPC < 0), and black indicates an insignificant trend (95% CI included 0).

### 3.2 Correlation with socio-demographic index

**Figure 1:**
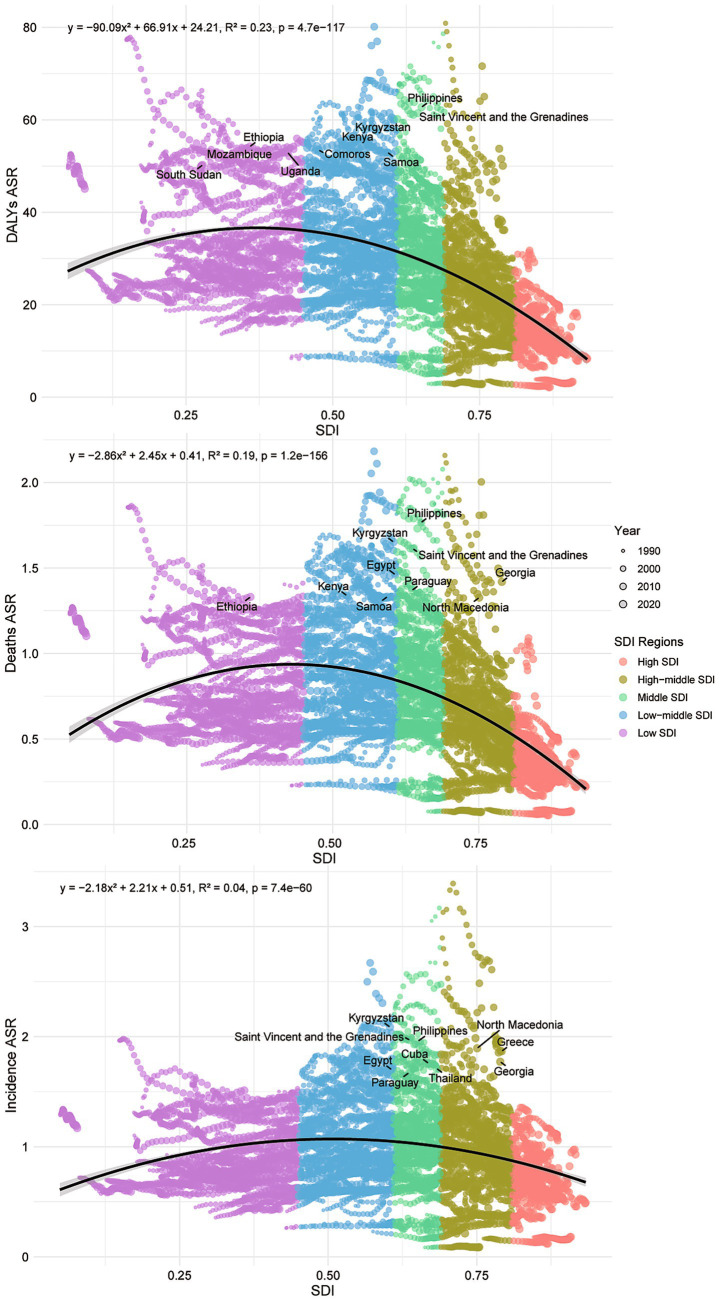
The scatter plot of polynomial regression analysis between SDI values and age-standardized DALYs, deaths, and incidence rate of MNBAC in 204 countries and territories from 1990 to 2021.

Incorporating data from 1990 to 2021, we observed a strong and statistically significant association between national SDI and disease burden indicators (ASIR, ASDR, and DALY ASR). Polynomial regression analysis revealed a predominantly inverse relationship, indicating that countries with higher socioeconomic development consistently experienced lower burden over the past three decades. Importantly, the relationship was not strictly linear: a nonlinear pattern emerged, suggesting that burden initially increased as SDI rose from low to middle levels but declined again at higher SDI levels, possibly reflecting improved diagnostic capacity followed by effective prevention and treatment. The heaviest burdens remained concentrated in the Philippines, Saint Vincent and the Grenadines, and Kyrgyzstan, which consistently ranked among the highest globally across all three indicators (Figure 1).

### 3.3 Age- and sex-specific burden

**Figure 2:**
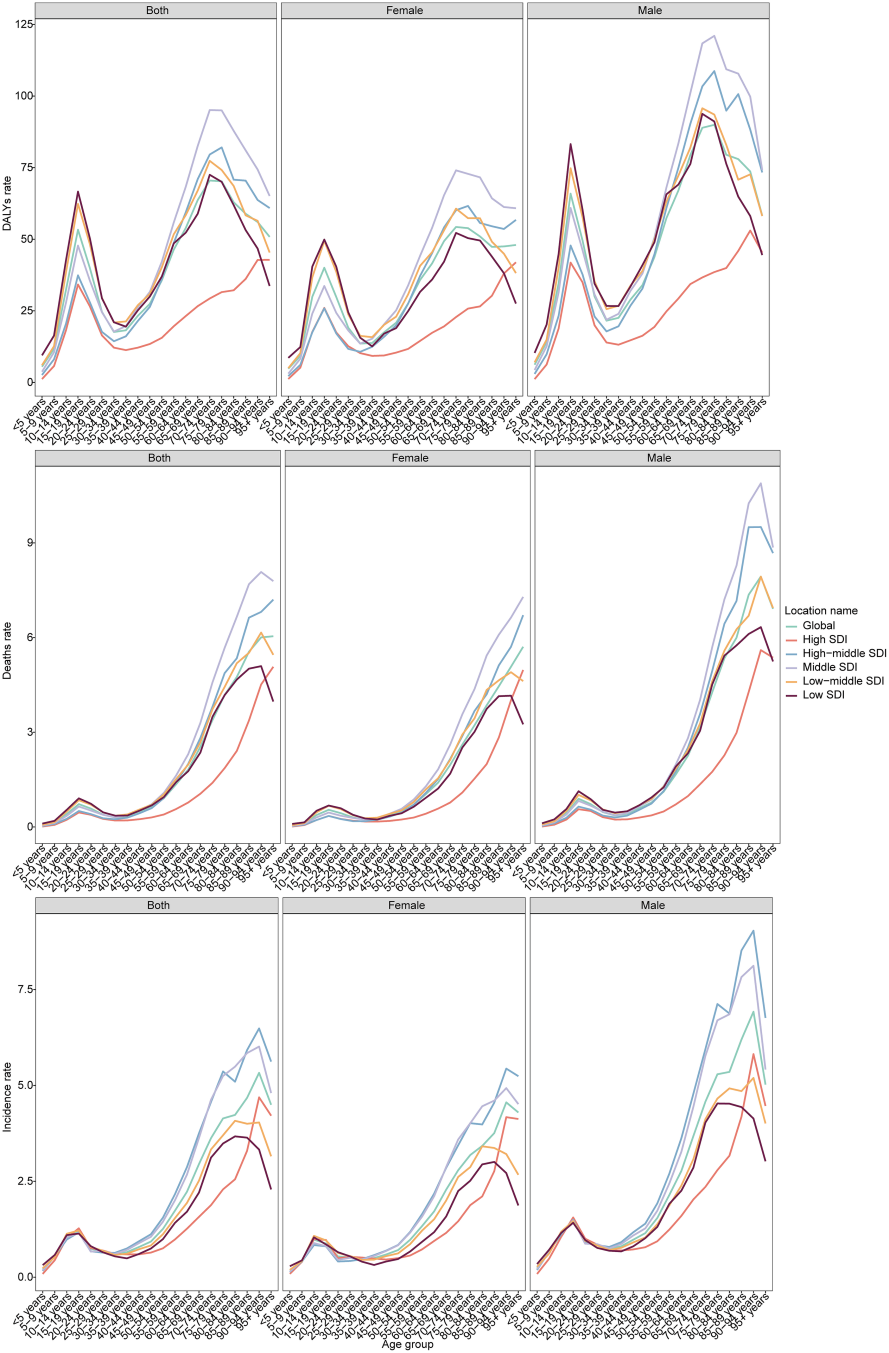
The DALYs, deaths, and incidence rate of MNBAC across different age groups and genders in global and 5 SDI regions in 2021.

Globally, the incidence rate increased before age 20, declined until 30–34 years, and rose again, peaking at ages 90–94 years (5.33 per 100,000; 95% CI: 4.10–6.29) (Figure 2). Among males, the peak incidence was 6.92 in ages 90–94 years. Among females, incidence peaked at 4.56 in ages 90–94 years, but also showed a distinct rise during adolescence. By SDI, the incidence was lowest in high SDI regions and highest in middle SDI regions. Mortality increased steadily with age, reaching 6.04 among individuals ≥95 years. Men showed the highest mortality at ages 90–94 years (7.93), while women peaked slightly later (≥95 years, 5.70). DALYs peaked earlier, at 70–74 years (70.50) globally, with men peaking at 75–79 years (89.95) and women at 70–74 years (54.28). The trends in MNBAC burden across different SDI regions vary significantly. Typically, low SDI regions may have persistently higher DALY rates, mortality rates, and incidence rates, while high SDI regions tend to have relatively lower rates. This reflects the correlation between socioeconomic status and health outcomes. In regions with better socioeconomic conditions, improved accessibility to healthcare resources, living environments, and health awareness may contribute to better overall health status.

### 3.4 Age-period-cohort effects

**Figure 3:**
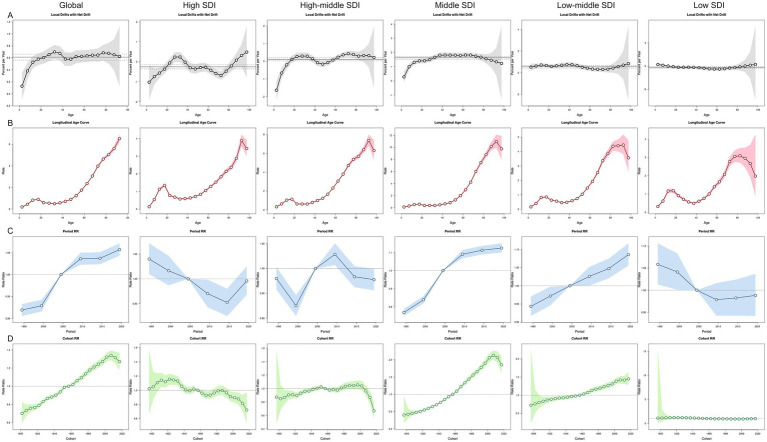
The effects of age, period, and birth cohort on the incidence of MNBAC in global and 5 SDI regions. **(A)** Local drift represents the annual percentage change of age-specific incidence rates in global and 5 SDI regions from 1992 to 2021 across 20 age groups (0–4 years to 95 + years). **(B)** Age effects are illustrated by the longitudinal age-specific incidence rates, adjusted for period bias and fitted for a specified number of birth cohorts. **(C)** Period effects are represented by the relative incidence risks (rate ratios), calculated as the ratio of age-specific incidence rates during the periods 1992–1996 and 2017–2021, with the reference period set as 2002–2006. **(D)** Birth cohort effects are shown by the relative risk of disease for birth cohorts, calculated as the ratio of age-specific incidence rates for the 1900–1904 cohort to the 2015–2019 cohort, with the reference cohort set as 1955–1959. Points and shaded areas represent incidence rates or rate ratios and their corresponding 95% confidence intervals. The black horizontal line represents the net drift value or the reference period.

Between 1992 and 2021, the global net drift for MNBAC incidence was 0.61% per year (95% CI: 0.56–0.66), with the fastest growth observed in ages 30–34 years (0.70%; 95% CI: 0.58–0.81) (Figure 3). High SDI and low SDI regions showed downward drifts (−0.25% and −0.31%, respectively), though high SDI regions saw localized increases in young adults (25–29 years). In contrast, middle SDI regions had the steepest increase (1.63%; 95% CI: 1.53–1.73). Cohort analysis revealed that individuals born after 1955 experienced progressively higher risks of MNBAC, especially in middle and low-middle SDI regions. In high SDI regions, risks declined overall despite minor fluctuations, while in middle-high SDI regions, incidence rose gradually before 2000 and then dropped among cohorts born after 2000.

### 3.5 Projections to 2030

**Figure 4:**
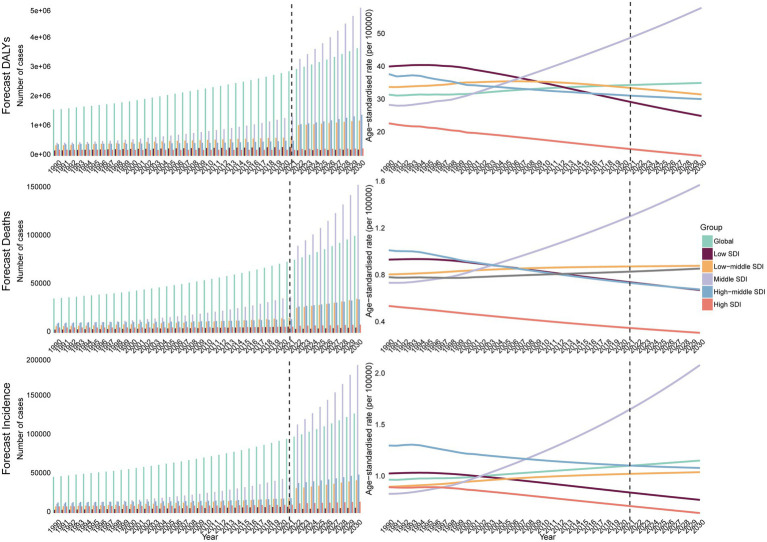
The BAPC model predicts the DALYs, deaths, incidence cases, and ASR of MNBAC from 2021 to 2030.

Bayesian forecasting indicated that by 2030, the global ASIR will reach 1.15, the ASDR 0.85, and the DALY ASR 34.8. The middle SDI region is projected to bear the steepest rise, with ASIR climbing to 2.08, ASDR to 1.57, and DALY ASR to 57.7 by 2030 (Figure 4). In contrast, other SDI regions are expected to remain stable or decline, except for a modest stabilization in low-middle SDI regions.

## 4 Discussion

This study leverages GBD 2021 estimates to provide a comprehensive, multi-level assessment of the global burden of malignant neoplasms of bone and articular cartilage (MNBAC) from 1990 to 2021, and projects burden through 2030 using Bayesian age-period-cohort modeling. By combining standardized measures (ASIR, ASDR, DALY ASR) with age-sex stratification, SDI-based comparisons, APC decomposition, and BAPC forecasting, our analysis delivers a detailed epidemiological picture that can inform prioritization and resource allocation for MNBAC control. We note that previous studies on MNBAC have primarily focused on regional disease burden, with most concentrating on China, the United States, and global-level analyses (13, 14). Although these studies have provided valuable insights into localized incidence patterns and short-term trends, they lack a global perspective and a SDI-stratified analysis, which is essential for understanding disparities across different levels of development. Our study fills this gap by conducting a comprehensive analysis across five SDI strata, revealing differences in disease burden among regions at varying developmental levels and identifying high-burden regions and populations that have been underemphasized in prior global MNBAC research.

Overall, we observed a substantial rise in absolute case counts, deaths, and DALYs between 1990 and 2021, with modest but statistically significant increases in global age-standardized rates. Regional and sociodemographic heterogeneity was evident: middle and low-middle SDI regions experienced the largest upward trends, whereas high-SDI regions generally showed declining or stable age-standardized rates. East and Southeast Asia accounted for some of the fastest regional increases in incidence, mortality, and DALY rates. These patterns suggest that the changing MNBAC burden is driven by a mixture of true epidemiological change and shifts in detection, diagnosis, and reporting capacity.

Several mechanisms plausibly underlie the observed increases. Improvements in health system capacity, cancer surveillance, and diagnostic technologies in many middle-income countries likely raised ascertainment and reporting of previously under-recognized cases. Concurrently, environmental exposures, changes in lifestyle and diet, demographic aging, and other contextual factors may have contributed to a genuine increase in disease incidence in some settings (15–17). The net effect is complex and likely non-linear: at low levels of development, limited diagnostic capacity can lead to under-ascertainment; as SDI rises to the middle range, improved detection—in combination with environmental and lifestyle changes—may increase observed burden; at the highest SDI levels, stronger prevention, early detection, and treatment systems may reduce age-standardized rates.

Our correlation analyses reflect this complexity. The polynomial regression model further confirmed a significant nonlinear relationship between SDI and MNBAC burden over 1990–2021. Rather than a simple monotonic decline, the association followed an inverted U-shaped pattern: disease burden initially increased as SDI rose from low to middle levels, but declined again in high-SDI settings. This pattern suggests that improvements in diagnostic capacity and shifts in risk exposure may temporarily increase disease detection in transitioning regions before comprehensive prevention and treatment systems reduce burden in highly developed contexts. These results underscore the importance of accounting for nonlinearity and temporal dynamics when interpreting SDI-burden associations.

Age- and sex-specific analyses reaffirmed well-recognized epidemiological features of MNBAC: a bimodal age distribution with peaks in adolescence and in older age, and a consistently higher burden among males. The adolescent peak emphasizes the importance of awareness and diagnostic vigilance in pediatric and adolescent health services, while the later-life peak underscores the need for geriatric oncology capacity and palliative care resources. Male predominance may reflect differential exposure to behavioral (e.g., tobacco, alcohol), occupational, and possibly hormonal or biological risk factors; however, the drivers likely vary by region and warrant targeted etiologic research (18–22).

By 2030, MNBAC incidence is expected to keep climbing in middle-SDI settings while plateauing or falling elsewhere, prompting a resource-realistic response: (1) strengthen pathology accuracy through periodic histotype workshops and telepathology backed by wider MRI/CT access and uniform staging; (2) embed lean, three-person sarcoma MDTs that deliver limb-sparing surgery and affordable generic chemotherapy; and (3) create regional sarcoma hubs feeding primary/secondary facilities and deploy community health workers for pain control, collectively curbing misdiagnosis, cost, and inequity to offset the rising burden.

This study has several strengths. It uses standardized GBD metrics to enable comparable estimates across 204 locations and three decades; it applies APC methods to separate age, period, and cohort effects; and it provides short-term forecasts to inform planning. At the same time, important limitations must be acknowledged. First, our analysis relies on GBD modeled estimates rather than primary, patient-level registry data. Misclassification, incomplete reporting, and heterogeneity in diagnostic practices can influence GBD outputs. Second, MNBAC is heterogeneous in histology, stage at diagnosis, and clinical course, but GBD aggregates these subtypes; subtype-specific burden (e.g., osteosarcoma, chondrosarcoma, Ewing sarcoma) could not be examined, limiting clinical interpretability. Third, important covariates—such as stage distribution, treatment access, and molecular or occupational exposures—are not captured in GBD and therefore cannot be directly evaluated. Fourth, forecasting with BAPC assumes that recent trends continue under current conditions; unexpected shifts in risk exposures, diagnostics, or therapeutics could materially alter future burden. Finally, under-ascertainment in low-resource settings remains a concern and may bias comparisons.

In light of these findings and limitations, we recommend the following priorities: (1) enhance cancer registry coverage and diagnostic capacity in middle and low-middle SDI regions to reduce under-ascertainment and facilitate subtype-specific surveillance; (2) invest in early detection and referral systems targeted at adolescents and older adults; (3) strengthen occupational and lifestyle risk-reduction programs, particularly where male predominance is observed; (4) expand access to multidisciplinary sarcoma care, including pathology, oncology, surgery, radiology, and rehabilitation; and (5) support prospective, population-based studies to clarify etiologic drivers and evaluate the impact of interventions.

In conclusion, while MNBAC remains a rare tumor group, its global impact has grown over the past three decades and is projected to rise further in several middle-SDI regions. Addressing this trend requires SDI-stratified, context-specific public health interventions, tailored to the healthcare capacities and needs of different regions. For middle-SDI regions—where the projected growth in MNBAC burden is most notable—priorities should include strengthening cancer registration systems (to improve the capture of MNBAC incidence, subtype distribution, and outcomes) and expanding access to essential diagnostic tools (such as imaging and pathological testing); these steps lay the foundation for accurate diagnosis and subsequent treatment planning. In low-SDI regions, interventions should first focus on building basic healthcare infrastructure—for example, establishing primary care facilities capable of recognizing early MNBAC-related symptoms—alongside rolling out cost-effective, population-based screening programs adapted to local resource constraints. These actions are critical to reducing delays in case identification. For high-SDI regions, which already have relatively mature healthcare systems, the focus should shift to optimizing early detection strategies (e.g., refining risk stratification for high-risk populations to enhance screening efficiency) and promoting multidisciplinary care models that integrate oncology, surgery, radiology, and palliative care. Such efforts will further improve treatment outcomes and quality of life for MNBAC patients. Collectively, these targeted measures will help mitigate the growing global burden of MNBAC—particularly in regions facing the greatest challenges or projected increases in disease burden.

## Data Availability

The original contributions presented in the study are included in the article/supplementary material, further inquiries can be directed to the corresponding author.

## Footnotes

^1^ https://analysistools.cancer.gov/apc/
